# A Spanish value set for the SF-6D based on the SF-12 v1

**DOI:** 10.1007/s10198-023-01657-9

**Published:** 2024-02-01

**Authors:** Jorge-Eduardo Martínez-Pérez, José-María Abellán-Perpiñán, Fernando-Ignacio Sánchez-Martínez, Juan-José Ruiz-López

**Affiliations:** Department of Applied Economics, University of Murcia, Murcia, Spain

**Keywords:** SF-6D, SF-12, Lottery equivalent methods, Value set, Social tariff, Preference-based measure, I10, I12

## Abstract

**Aim:**

This paper reports the first estimation of an SF-6D value set based on the SF-12 for Spain.

**Methods:**

A representative sample (n = 1020) of the Spanish general population valued a selection of 56 hypothetical SF-6D health states by means of a probability lottery equivalent (PLE) method. The value set was derived using both random effects and mean models estimated by ordinary least squares (OLS). The best model was chosen on the basis of its predictive ability assessed in terms of mean absolute error (MAE).

**Results:**

The model yielding the lowest MAE (0.075) was that based on main effects using OLS. Pain was the most significant dimension in predicting health state severity. Comparison with the previous SF-6D (SF-36) model estimated for Spain revealed no significant differences, with a similar MAE (0.081). Nevertheless, the new SF-6D (SF-12) model predicted higher utilities than those generated by the SF-6D (SF-36) scoring algorithm (minimum value − 0.071 vs − 0.357).

**Conclusion:**

A value set for the SF-6D (SF-12) based on Spanish general population preferences elicited by means of a PLE technique is successfully estimated. The new estimated SF-6D (SF-12) preference-based measure provides a valuable tool for researchers and policymakers to assess the cost-effectiveness of new health technologies in Spain.

## Introduction

Quality-adjusted life years (QALYs) are commonly recommended as the preferred outcome measure to be used in health technology assessment (HTA) [1], as they combine survival and health-related quality of life (HRQoL) into a single number [2]. Utility weights used to calculate QALY-based cost-effectiveness ratios can be obtained from health-related multiattribute utility (MAU) instruments, such as, among others, the EuroQol five-dimensions (EQ-5D), with three or five levels [3, 4], Assessment of Quality of Life [5], Health Utilities Index (HUI) 2 and 3 [6, 7], and the two versions of the six-dimensional health state Short Form (SF-6D) derived from the Short-Form 36 (SF-36) health survey [8, 9]. All these MAU instruments use value sets, also called tariffs, based on the preferences elicited from a representative sample of the general population by means of valuation techniques such as discrete choice experiment (DCE) [2, 10, 11], time trade-off (TTO) [2, 10, 12, 13], standard gamble (SG) [8], or lottery equivalent (LE) methods [14].

The SF-36 is a 36-item questionnaire [15] that covers eight health domains: physical functioning, role limitations due to physical health, role limitations due to emotional problems, energy/fatigue or vitality, emotional well-being or mental health, social functioning, bodily pain, and general health. There are two versions of the SF-36 [16, 17] that differ in the number of response choices on some items and the way that domain scores are interpreted. This health survey is commonly used in health status assessment and HTA [18, 19]. The SF-36 has been translated and validated in various languages, including Spanish, following a standardized procedure [20].

The SF-12 health survey is a shortened version of the SF-36 questionnaire, comprising 12 items. As occurred with the SF-36, there are also two versions of the SF-12 questionnaire [21, 22]. Existing evidence suggests that the SF-12 can replicate more than 90 percent of the variance in both physical and mental component summary scores of the SF-36 [22, 23], demonstrating reliability and validity as a shorter form of the questionnaire [21, 24].

Neither the SF-36 nor the SF-12 can yield utility weights by themselves, so they are not able to compute QALYs directly. Nevertheless, as SF-6D health states are based on SF-36/SF-12 items, value sets for the SF-6D allow analysts and practitioners to attach preference scores to SF-36/SF-12 surveys. In this way, the seminal study conducted by Brazier et al. [8] estimated the SF-6D value set from the SF-36 for the UK. Later on, Brazier and Roberts [25] inferred the SF-6D value set from the SF-12 using the same data set of the previous study [8].

In contrast to the UK, there is no SF-6D value set derived from the SF-12 for Spain; there is only one SF-6D tariff based on the SF-36 [14]. This article aims to address this gap by presenting a new SF-6D value set derived from the SF-12 and comparing it with the previous one based on the SF-36, both originated from the same survey, similar to what Brazier and Roberts [25] did for the UK.

The paper is organized as follows. The next section describes the design of the valuation survey, as well as the health states selected, the elicitation procedure applied to value them, and the modeling strategy used to estimate the SF-6D (SF-12) value set. Section “Results” shows the main results of our study, including the model finally chosen. The estimated value set is compared to that previously estimated from the SF-36 in Spain and to the SF-6D (SF-12) UK’s tariff as well. Finally, “Discussion” closes the paper.

## Methods

### Study design

The valuation survey was designed to value health states defined by the SF-6D (SF-36), and the data from this survey are used in this study to estimate a preference-based scoring algorithm for the SF-6D (SF-12). This version of the SF-6D is shown in Table 1.

**Table 1:** The SF-6D (the SF-12 version)

Physical functioning (PF)		Pain (PAIN)	
PF1	Your health does not limit your moderate activities	PAIN1	You have pain but it does not interfere with your normal work (both outside the home and housework) at all
PF2	Your health limits you a little in moderate activities	PAIN 2	You have pain that interferes with your normal work (both outside the home and housework) a little bit
PF3	Your health limits you a lot in moderate activities	PAIN 3	You have pain that interferes with your normal work (both outside the home and housework) moderately
		PAIN 4	You have pain that interferes with your normal work (both outside the home and housework) quite a bit
		PAIN 5	You have pain that interferes with your normal work (both outside the home and housework) extremely
Role limitations (RL)		Mental health (MH)	
RL1	You have no problems with your work or other regular daily activities as a result of your physical health or any emotional problems	MH1	You feel downhearted and low none of the time
RL2	You are limited in the kind of work or other activities as a result of your physical health	MH2	You feel downhearted and low a little of the time
RL3	You accomplish less than you would like as a result of emotional problems	MH3	You feel downhearted and low some of the time
RL4	You are limited in the kind of work or other activities as a result of your physical health and accomplish less than you would like as a result of emotional problems	MH4	You feel downhearted and low most of the time
		MH5	You feel downhearted and low all of the time
Social functioning (SF)		Vitality (VIT)	
SF1	Your health limits your social activities none of the time	VIT1	You have a lot of energy all of the time
SF2	Your health limits your social activities a little of the time	VIT2	You have a lot of energy most of the time
SF3	Your health limits your social activities some of the time	VIT3	You have a lot of energy some of the time
SF4	Your health limits your social activities most of the time	VIT4	You have a lot of energy a little of the time
SF5	Your health limits your social activities all of the time	VIT5	You have a lot of energy none of the time

A representative sample of the general Spanish population (n = 1020) was obtained through a two-stage stratified sampling methodology. To optimize the response rate, recruitment strategies included advance contact, reminders, appointment scheduling, and small gifts. Since the survey was sponsored by the Department of Health, high collaboration was achieved, obtaining a response rate of 90%. The survey received approval from the ethics committee. Respondents were interviewed and grouped into 17 subsamples, consisting of 60 individuals each. Each respondent valued a maximum of 5 health states out of a total of 78 using a probability lottery equivalent (PLE) method. The detailed design of the survey has been reported elsewhere [14].

### Selection of health states

As noted above, a total of 78 SF-6D (SF-36) health states were used in the survey. Of these, 49 were obtained by running the Orthoplan module of SPSS version 17, which yields the minimum subset of states, which allows the estimation of an additive model. The remaining states (including the worst possible SF-6D state, the so-called ‘pits’ state) up to 78 were included to estimate potential interaction effects between attributes. This subset of 78 health states, originally selected for the estimation of the SF-6D (SF-36) tariff [14], was reduced to 56 states (Table 2) to estimate the SF-6D (SF-12) algorithm. Since the PF and PAIN dimensions have a more reduced number of levels in the SF-12-based version of the SF-6D than in the SF-36-based one, some states were excluded from the estimation.

**Table 2:** The SF-6D (SF-12) health states valued

111113	112351	121522	233333	143443	323333	312322
111115	113131	122122	233533	144311	323414	322111
111131	113515	122155	235144	145254	331253	124535
111311	115111	133222	242523	211112	334445	132512
122232	115433	134143	243233	214245	334531	142441
123434	121425	135121	244325	222134	344145	233121
124123	131324	135142	311111	225312	344444	231451
124152	132144	141214	311142	225454	345125	345254

In the initial setup, all respondents were required to assess 5 health states, which were randomly distributed across the 17 models from the original pool of 78 states. Consequently, some health states were evaluated in more than one model, leading to a higher number of valuations. As a consequence of reducing the number of health states from 78 to 56 compatible with the SF-12, the range of health states valued by each individual considered for modeling varies between 2 and 5.

### The interviews

Data were collected through face-to-face computer-assisted personal interviews (CAPI) conducted by trained interviewers. The average duration per interview was around 20 min. The survey was divided into four parts. First, the SF-6D classification system was explained to the respondents. Second, they were asked to rate five SF-6D health states on a visual analogue scale. Next, preference weights for the five health states were elicited using the PLE. Specifically, information was gathered concerning gender, marital status, highest level of education attained, monthly income, and health habits, such as whether the interviewee was a smoker or not. Regarding health status, respondents fulfilled both EQ-5D-3L and SF-12 instruments.

### Valuation method

The PLE asks for the probability *p* that makes the respondents indifferent between the gamble denoted by (full health, *p*; death), yielding full health with probability *p* and death with probability 1–*p*, and the gamble denoted by (full health, 0.5; *h*), yielding full health and the health state *h* with the same probability. This approach enabled us to capture preferences for states regarded as both better and worse than death. If the respondent favored the second gamble over the first in the initial question (i.e., for p = 0.5), it indicated that state h was perceived as preferable to death. Consequently, the ultimate probability of indifference, denoted as p*, was elicited within the range of 0.5 to 1. Conversely, if the first gamble was preferred over the second for p = 0.5, then state h was considered worse than death, and p* was elicited within the range of 0 to 0.5. Following the expected utility theory, assuming the convention that the utility of perfect health is 1 and the utility of death is 0, the utility of health state h is calculated as U(h) = 2p* – 1.

### Modeling

Our initial specification is a model without interactions between variables, that is, a main effects model, whose constant term was forced to unity to ensure that the utility of full health equals one. In our analysis, we introduced a dichotomous variable labeled 'MOST,' taking a value of 1 if any dimension reached its maximum level and 0 otherwise. We also introduced a variable aiming to capture the total sum of dimensions within the state. However, these variables did not improve the model in any case, leading to their exclusion from the final specification. The model was estimated by following a dual regression approach, as common in many studies [8, 26–29], using both ordinary least squares (OLS) and random effects (RE) estimators. The optimal model is chosen on the basis of its predictive ability in terms of mean absolute error (MAE) and the proportion of predictions outside 0.01, 0.05, and 0.1 ranges on either side of the actual value.

## Results

### The sample

A total of 15 participants were excluded from the analysis due to ordinal inconsistencies in their PLE valuations. Such inconsistencies came from the fact that some health states could be logically ordered, i.e., one of them had an equal or higher (worse) level than the other one in each of the six dimensions. An ordinal inconsistency occurred when a higher value was assigned to a logically more severe health state than to a less severe one. Furthermore, seven respondents were excluded from the analysis because they were not willing to accept any risk of death to improve their health, thus implicitly assigning a utility 1 to a health state which is worse than perfect health. After exclusions, the final sample used to estimate the SF-6D (SF-12) scoring algorithm consisted of 998 individuals. This exclusion rate (2.16%) is slightly lower than that observed in the Dutch study [31], which was 2.5%. Table 3 shows the sociodemographic characteristics of this sample. Compared to the general Spanish population, some differences arise in terms of educational achievements and income earnings.

**Table 3:** Sociodemographic characteristics of included respondents and Spanish population

	Sample (*n* = 998)	Spanish population
*Male/Female (%)*	50/50	51/49
*Mean (SD) age in years*	43.6 (16.64)	42.7 (16.9)
*Marital Statuts*		
Single	33.7	32.4
Married/cohabiting	59.8	63.1
Separated/divorced/widow	6.5	4.5
*Education Level*		
Illiterate/primary studies	34.5	30.1
Secondary studies	34.4	45.1
University studies	31.1	24.7
*Income Level*		
Up to €2000	51.3	69.5
€2000-3000	29.8	19.5
More than €3000	18.9	11
*Smoker (%)*	27	29.8
*Self-assessed health state (EQ-5D)*		
11111	60.8	
11121	15.8	
11112	4.3	
Other	19.1	
*Self-assessed health state (SF-6D)(SF-12)*		
111122	9.1	
111112	4.3	
111121	1.5	
111111	2.9	
Other	82.2	

### The data set

Each of the 56 health states presented in Table 4 was assessed an average of 64 times, with a minimum of 56 participants and a maximum of 119 participants providing valuations. Mean values range from 0.139 to 0.988, exhibiting substantial standard deviations. Notably, the distribution of intended sample sizes for valuation is bimodal—60 for a subset of health states and 120 for the remainder. Consequently, relying exclusively on mean values may not adequately capture the full distributional properties of the data. The utility scores themselves demonstrate non-normal distributional attributes, as characterized by a negative skewness of -0.840 and elevated kurtosis of 5.077, indicative of a platykurtic and left-skewed distribution. The median utility score stands at 0.6, and the 25th and 75th percentiles are 0.38 and 0.8, respectively. Notably, no median values below zero were observed, which aligns with findings reported by Brazier and Roberts [25]. Approximately, 23% of the health states received negative utility values from at least one participant, contrasting with Brazier and Roberts' study, which reported a lower occurrence of negative utilities [25].

**Table 4:** Statistics for SF-6D (SF-12) health state valuations

State	Mean	SD	Median	Min	Max	*n*
111113	0.903	0.146	0.960	0.200	1.000	59
111115	0.855	0.197	0.900	− 0.960	1.000	114
111131	0.878	0.110	0.900	0.500	1.000	120
111311	0.803	0.105	0.780	0.540	1.000	119
112351	0.515	0.137	0.500	0.200	0.800	60
113131	0.988	0.036	1.000	0.800	1.000	58
113515	0.449	0.254	0.400	− 0.300	0.900	58
115111	0.649	0.122	0.660	0.300	0.800	118
115433	0.383	0.298	0.400	− 0.900	0.960	57
121425	0.569	0.145	0.600	0.160	0.900	60
121522	0.451	0.103	0.460	0.180	0.700	60
122122	0.891	0.130	0.930	0.520	1.000	60
122155	0.469	0.167	0.460	0.200	0.900	59
122232	0.826	0.136	0.820	0.400	1.000	58
123434	0.474	0.190	0.420	0.060	0.820	56
124123	0.760	0.172	0.800	0.200	1.000	60
124152	0.411	0.158	0.400	0.100	0.940	60
124535	0.297	0.159	0.260	0.060	0.700	60
131324	0.340	0.102	0.300	0.160	0.680	60
132144	0.605	0.136	0.600	0.320	0.900	59
132512	0.710	0.174	0.700	0.300	1.000	59
133222	0.671	0.136	0.660	0.360	1.000	59
134143	0.381	0.114	0.360	0.180	0.620	59
135121	0.610	0.134	0.600	0.400	1.000	59
135142	0.595	0.170	0.600	0.100	0.900	56
141214	0.755	0.176	0.800	0.240	1.000	56
142441	0.613	0.193	0.560	0.300	1.000	59
143443	0.400	0.125	0.380	0.160	0.740	59
144311	0.675	0.234	0.700	− 0.200	1.000	59
145254	0.375	0.161	0.400	0.060	0.700	56
211112	0.908	0.089	0.940	0.580	1.000	57
214245	0.395	0.122	0.400	0.200	0.800	59
222134	0.598	0.211	0.600	0.000	1.000	60
225312	0.552	0.182	0.510	0.000	0.900	58
225454	0.191	0.323	0.200	− 0.900	0.700	58
231451	0.522	0.358	0.560	− 0.800	0.960	59
233121	0.865	0.296	0.980	− 0.940	1.000	58
233333	0.490	0.174	0.500	0.100	0.900	60
233533	0.514	0.184	0.480	0.200	0.900	59
235144	0.557	0.329	0.640	− 0.600	0.980	59
242523	0.227	0.516	0.200	− 0.980	1.000	58
243233	0.443	0.151	0.420	0.200	0.800	60
244325	0.160	0.085	0.160	0.060	0.580	60
311111	0.895	0.100	0.900	0.600	1.000	117
311142	0.890	0.127	0.900	0.180	1.000	56
312322	0.599	0.178	0.600	0.300	1.000	60
322111	0.757	0.099	0.760	0.300	0.860	60
323333	0.458	0.077	0.460	0.340	0.680	60
323414	0.138	0.149	0.100	− 0.200	0.600	59
331253	0.584	0.156	0.600	0.200	0.900	56
334445	0.241	0.102	0.220	0.060	0.520	59
334531	0.153	0.304	0.200	− 0.880	0.780	60
344145	0.260	0.251	0.200	− 0.400	0.900	58
344444	0.416	0.206	0.400	0.080	0.940	59
345125	0.369	0.140	0.330	0.100	0.800	60
345254	0.265	0.466	0.400	− 0.980	0.920	59

**Fig. 1:**
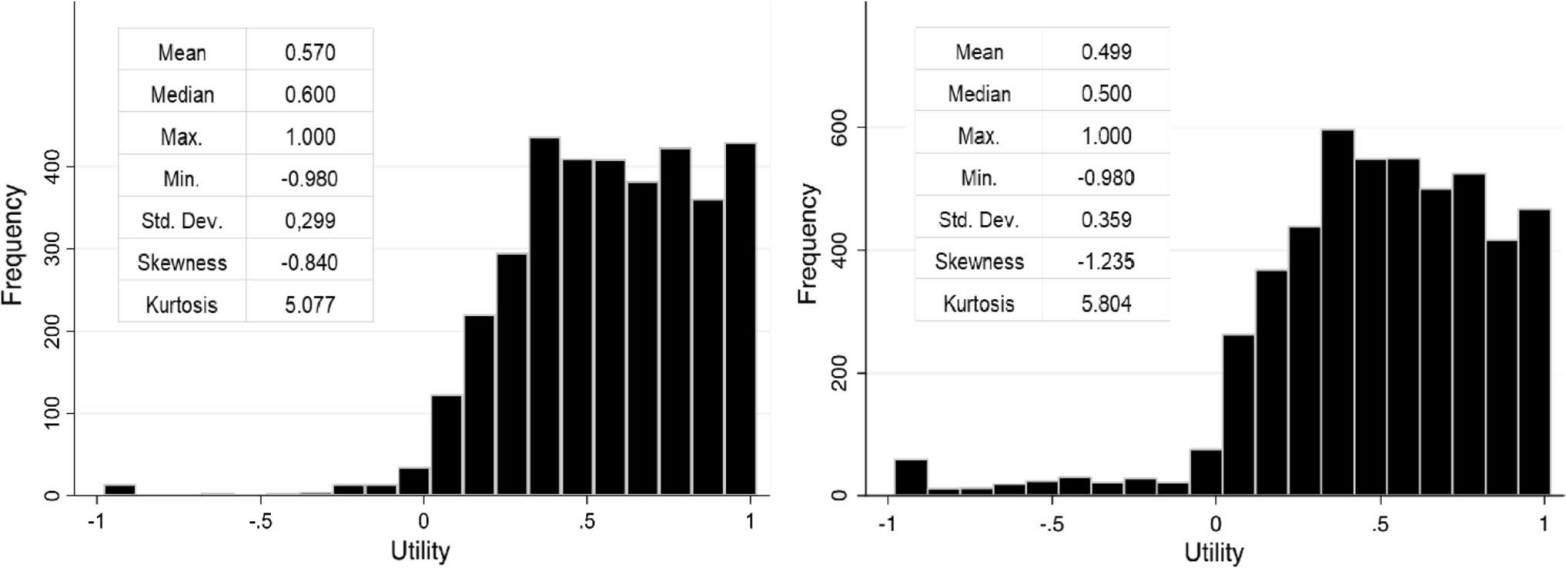
Utility histogram of SF-6D (SF-12) and SF-6D (SF-36)

Figure 1 completes the picture shown in Table 4. It displays a histogram and some descriptive statistics for the whole distribution of SF-6D (SF-12) individual valuations.

Table 5 shows coefficients estimated for five models. All the models predict SF-6D (SF-12) utilities except for model (5) which is based on the SF-36 [14]. Models (1) and (2) are RE models, while models (3), (4), and (5) are OLS models using mean values with corrective weights proportional to the number of individuals valuing each health state. ‘Raw models’ are models without the removal of non-significant variables, whereas ‘efficient models’ were constructed by eliminating non-significant regressors from ‘raw models’ and by grouping the variables of any two consecutive levels when their coefficients are not significantly different from each other according to the value of the Wald statistic. We did not find any significant interaction term, so all our algorithms only reflect the main effects.

**Table 5:** Comparing SF-6D (SF-12) and SF-6D (SF-36) algorithms

SF-6D(SF-12)								SF-6D(SF-36)	
	RE model				OLS mean model			OLS mean model	
	(1) Raw		(2) Efficient		(3) Raw		(4) Efficient		(5) Efficient
PF2	− 0.052***	PF2	− 0.063***	PF2	− 0.035***	PF2	− 0.040***	PF2	− 0.015***
PF3	− 0.095***	PF3	− 0.104***	PF3	− 0.098***	PF3	− 0.096***	PF3	− 0.034***
								PF4	− 0.090***
								PF5	− 0.111***
								PF6	− 0.338***
RL2	0.008			RL2	− 0.046***			RL2	− 0.014***
RL3	− 0.050***	RL23	− 0.025***	RL3	− 0.043***	RL23	− 0.044***	RL3	− 0.038***
RL4	− 0.075***	RL4	− 0.095***	RL4	− 0.057***	RL4	− 0.084***	RL4	− 0.070***
SF2	− 0.107***	SF2	− 0.069***	SF2	− 0.066***	SF2	− 0.048***	SF2	− 0.037***
SF3	− 0.105***	SF3	− 0.113***	SF3	− 0.099***	SF3	− 0.108***	SF3	− 0.060***
SF4	− 0.241***	SF4	− 0.214***	SF4	− 0.209***	SF4	− 0.207***	SF4	− 0.203***
SF5	− 0.273***	SF5	− 0.251***	SF5	− 0.230***	SF5	− 0.219***	SF5	− 0.208***
PAIN2	− 0.024**	PAIN2	− 0.020*	PAIN2	− 0.049***	PAIN2	− 0.031***	PAIN2	− 0.018***
PAIN3	− 0.113***	PAIN3	− 0.133***	PAIN3	− 0.191***			PAIN3	− 0.034***
PAIN4	− 0.218***	PAIN4	− 0.205***	PAIN4	− 0.184***	PAIN34	− 0.186***	PAIN4	− 0.198***
PAIN5	− 0.304***	PAIN5	− 0.312***	PAIN5	− 0.300***	PAIN5	− 0.291***	PAIN5	− 0.202***
								PAIN6	− 0.318***
MH2	− 0.035***	MH2	− 0.044***	MH2	− 0.092***			MH2	− 0.066***
MH3	− 0.014	MH3	− 0.056***	MH3	− 0.056***	MH23	− 0.080***	MH3	− 0.078***
MH4	− 0.033**	MH4	− 0.061***	MH4	− 0.092***	MH4	− 0.090***	MH4	− 0.096***
MH5	− 0.170***	MH5	− 0.196***	MH5	− 0.198***	MH5	− 0.208***	MH5	− 0.224***
VIT2	− 0.019*			VIT2	0.004			VIT2	− 0.058***
VIT3	− 0.135***	VIT23	− 0.060***	VIT3	− 0.107***	VIT23	− 0.053***	VIT3	− 0.121***
VIT4	− 0.179***			VIT4	− 0.159***	VIT4	− 0.154***	VIT4	− 0.157***
VIT5	− 0.162***	VIT45	− 0.156***	VIT5	− 0.174***	VIT5	− 0.173***	VIT5	− 0.199***
Predictive ability									
MAE	0.083		0.083		0.076		0.075		0.081
|pred.error|<									
k(%)									
k=0.01	4.96		6.58		9.73		9.82		11.72
k=0.05	43.97		32.45		45.73		42.43		36.49
k=0.10	66.90		67.10		68.66		70.39		70.05

*MAE* mean absolute error

***p<0.01, **p<0.05, *p<0.1

The lowest MAE among the five models corresponds to model (4), which is slightly lower (0.075) than that (0.079) attached to the recommended SF-6D (SF-12) model for the UK [25]. Notwithstanding, all five models exhibit small, unbiased, and normally distributed MAE values. When comparing models (4) and (5) each other, RL and SF dimensions are weighted heavier in the former rather than the latter one. Across all five models, PAIN appears to be the most influential dimension in determining health state values. The PF dimension has a greater importance in generating health state values in the SF-6D (SF-36) algorithm for Spain but loses relevance in all the SF-6D (SF-12) models.

The results presented in Table 5 can be used to estimate utility weights for each health state. For example, the estimated value for state 344444 is 0.183 according to the SF-6D (SF-12) model 4 (calculated as 1–0.096–0.084–0.207–0.186–0.090–0.154), and 0.242 from the SF-6D (SF-36) model 5.

In comparing the utility weights assigned to each health state according to the SF-6D (SF-36) and SF-6D (SF-12) frameworks (Fig. 1), a higher prevalence of utilities falling below zero is discernible in the SF-6D (SF-36) version [14]. Although both value sets display negative skewness, this is more accentuated in the SF-6D (SF-12) version, which also records a higher frequency of utilities equating to unity. Notably, while both utility distributions share identical minimum and maximum values, the SF-6D (SF-12) value set registers higher median and mean values.

**Fig. 2:**
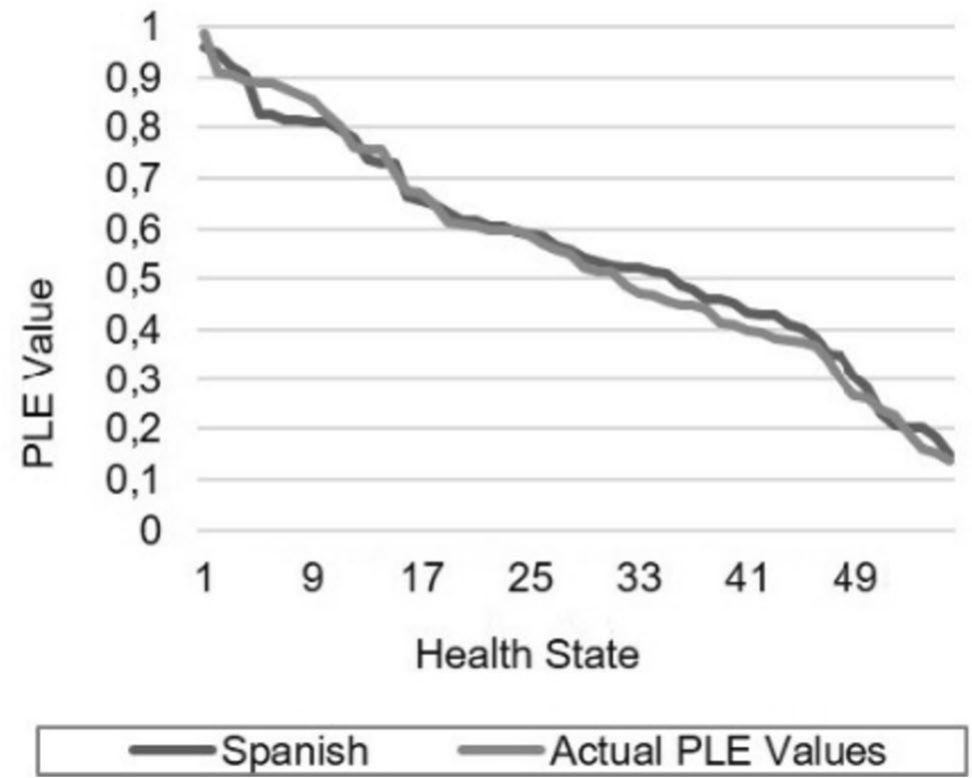
Plot of actual and predicted values for model 4

**Fig. 3:**
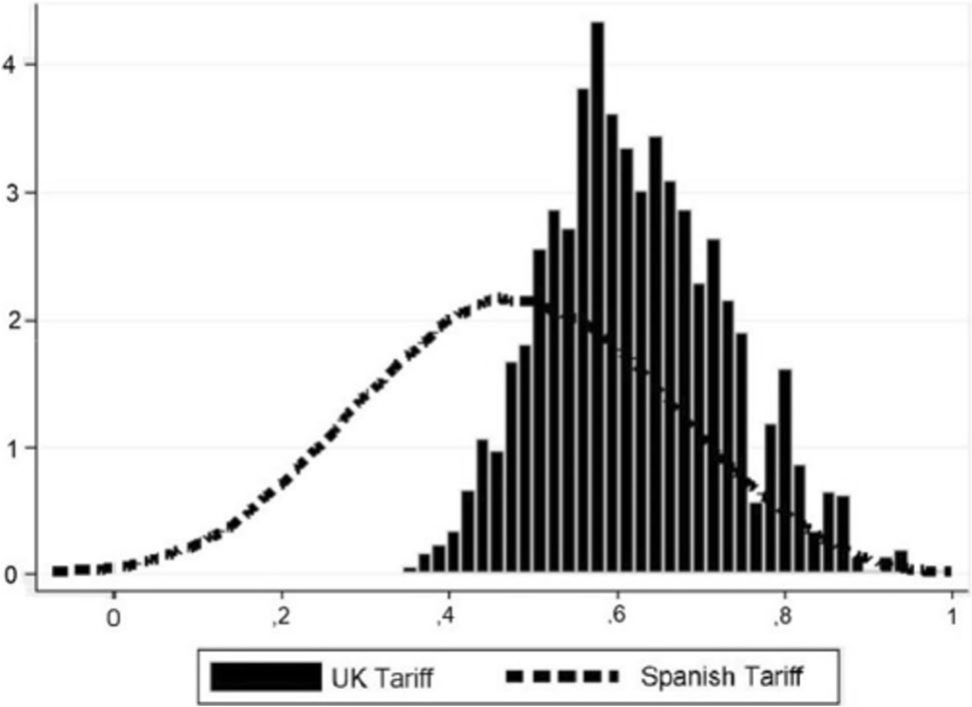
A comparison of the SF-6D (SF-12) Spanish and UK tariffs’ predicted values

Figure 2 illustrates the strong correlation between the utilities predicted by our preferred model (model 4) and those directly elicited from the respondents by the PLE for the same health states. In contrast to the Brazier and Roberts’ model for the SF-6D (SF-12) (see Fig. 1 in their article [25]) our model does not tend to overpredict the value for poor health states as their model does. This fact suggests that the Spanish tariff is not affected by floor effects. The conclusion is further substantiated by Fig. 3, which demonstrates that the Spanish tariff yields lower utility values in comparison to its UK counterpart.

## Discussion

This paper provides the first estimation of an SF-6D (SF-12) tariff ever done for Spain. It adds to the unique two previous estimations of the SF-6D value set from the SF-12 reported elsewhere [30] for the British [25] and Dutch [31] populations. The estimates reported in this article will allow analysts and practitioners to attach utility weights to patients’ health states described in the SF-12 survey.

The approach followed in the study reported in this paper to estimate an SF-6D (SF-12) value set is similar to that carried out in the UK [25]. In both cases, the valuation survey was designed to value health states defined by the SF-6D (SF-36), and the data from these surveys were used for the estimation of the SF-6D tariff from the SF-12. In addition, regression approaches used in the two studies are also quite similar. Differences arise between the two studies, however, in the set of health states selected, the number of them valued by each respondent, and the sample size. Brazier and Roberts [25] used a set of 249 health states, over four times our selection of 56 states. Respondents of our larger sample (998 vs 611), in return, valued a maximum of 5 health states each, one less than the number valued by each participant in the UK’s sample. Nevertheless, the biggest difference between both studies is due to the valuation technique used to value the health states. Whereas in the British study [25] valuations were elicited by means of the SG, a PLE method was used in ours.

This later difference is relevant because the distribution of predicted values by the model recommended in this paper extends utilities over a wider range than the UK tariff, ‘lowering’ the SF-6D (SF-12) floor. This mitigation of floor effects is due, as explained with regard to the SF-6D (SF-36) by Abellán-Perpiñán et al. [14], to the LE method used [32], i.e., the PLE, avoids the so-called ‘certainty effect’ (34), a bias that distorts SG measurements. Otherwise, the SF-6D (SF-12) model recommended in this paper has a slightly better predictive ability than Brazier and Roberts’ [25] preferred model while it does not tend to overpredict poor health states. Our tariff outperforms the UK’s one in terms of a closer correlation between predicted and actual valuations.

On the contrary, our study is not easily comparable to Jonker et al. [31] because both empirical designs are very different. The aim of the Jonker et al.’s [31] study is to estimate a time-preference corrected QALY tariff, the reason why they combine SF-6D (SF-12) health states with different life years in the pairwise choices that respondents have to make. The inclusion of life duration as an additional attribute allows the authors to accommodate nonlinear time preferences in DCE studies. As Jonker et al. [31] argue the assumption of linear time preferences can explain, at least partially, the relatively low QALY values usually reported in DCE duration studies compared to those based on TTO. It has to be noted, however, that in contrast to the TTO the PLE method used in our study is free from utility curvature biases, so our utilities do not have to be corrected to prevent that bias.

The ranking in terms of the importance of different dimensions is known to have cultural roots, and significant differences have been observed between countries and cultures (Wang and Poder (2023)) [30]. The order obtained in this study is not markedly different from that observed in previous studies for SF-6D (SF-12). Indeed, both the British study, with a ranking headed by PAIN and followed by MH, SF, VIT, PF, RL, and the Dutch study, with a ranking described by PAIN, MH, VIT, SF, PF, RL, show similarities to the one obtained in this study (PAIN, SF, MH, VIT, PF, RL). As in the two previous studies, PAIN and MH play a prominent role, while PF and RL occupy the lower positions. The comparison of the estimated tariff in this article with the Spanish tariff of the SF-6D (SF-36) [14] highlights the shifting impact on health dimensions. Indeed, in the SF-6D (SF-36) algorithm, it was PF the dimension that could potentially lead to the highest decrease in utility, whereas in the one presented here, it is PAIN the dimension placing such a position. In our view, this diminishing significance is primarily explained by the alteration in the instrument's structure, particularly the reduction from 6 to 3 levels in the PF dimension.

A limitation of this study resides in the sample size. It is important to acknowledge that our sample size of 1020 participants falls slightly below the optimal threshold for achieving full representativeness, as a minimum of 1,067 participants is typically required for a representative sample with 95% confidence and a 3% margin of error. Nonetheless, the sample size used in this study is clearly larger than the one used for the study in the UK [25], but smaller than that of the study conducted in the Netherlands [31]. Another limitation of this work is that the database used was generated more than a decade ago [14], however, it does not seem an unrealistic assumption to think that preferences for health states enjoy some temporal stability. Indeed, the best proof of this is that the tariffs of Brazier et al. [8] or Dolan [12] continue to be used, despite being substantially older. Another limitation of this study pertains to the slight discrepancies in income and educational level between our sample and the adult Spanish population. This constraint could be addressed in a manner akin to the approach employed by Méndez et al. [34]. However, in the interest of facilitating comparability with the extant tariff calculated using the same dataset [14], we have opted for an analogous analysis.

The survey did not incorporate any tasks to assess the numerical skills of the participants, which is unfortunate. This represents a limitation of the study, as elicitation through Probability-Lottery Equivalent (PLE) is a complex task that involves risk communication. Nevertheless, the survey did include some explanations about the risk and how to articulate it. Effective risk communication strategies described in the literature were employed: visual aids were utilized for risk communication [35] and the risks were presented as natural frequencies, a format known to enhance understanding [36]

A natural extension of this work involves estimating the SF-6Dv2 [9] tariff for the Spanish population. The SF-6Dv2 score is derived from 10 items of the SF-36v2. Compared to the SF-6Dv1, the SF-6Dv2 delineates more discrete health levels and diminishes floor effects. To the best of our knowledge, a tariff for SF-6Dv2 has been estimated only for a limited number of countries: UK [37], China [38], and Australia [11]

The availability of an SF-6D (SF-12) value set free from floor effects as that reported in this paper offers at least two advantages. First, it ensures that the instrument is sensitive enough to capture even the poorest health states, allowing for accurate assessment of severe health conditions and disabilities. Furthermore, a floor-free SF-6D tariff from the SF-12 enables comprehensive and reliable comparisons of health outcomes across different populations and interventions. This facilitates unbiased cost-effectiveness analyses and, hence, a more efficient resource allocation.

## Data Availability

The data that support the findings of this study are available from the corresponding author upon reasonable request.
